# Clinical outcomes after use of checkpoint inhibitor immunotherapies in people with multiple sclerosis

**DOI:** 10.1177/20552173241252563

**Published:** 2024-05-07

**Authors:** Alyssa N Nylander, William Rowles, Shane Poole, Riley Bove

**Affiliations:** 8785 University of California San Francisco, Weill Institute for Neurosciences, UCSF, San Francisco, CA, USA

**Keywords:** Retrospective studies, neoplasms, immune checkpoint inhibitors, immunotherapy, multiple sclerosis, progressive multifocal leukoencephalopathy

## Abstract

**Background:**

Immune checkpoint inhibitors (ICIs) represent a novel class of agents approved for the treatment of several cancers and progressive multifocal leukoencephalopathy (PML). However, due to the risk of autoimmune side effects, their use in people with autoimmune diseases such as multiple sclerosis (MS) has been limited.

**Objective:**

To characterize outcomes in a cohort of adults with MS who received ICIs.

**Methods:**

A single-center retrospective review of medical record data was performed for people with MS treated with ICIs.

**Results:**

Seven people with MS were identified, with a mean (SD) age at ICI use of 55.4 (13.7) years and a mean MS duration of 18.2 (12.2) years. Six were treated for cancer; 1 was treated for PML. After mean (SD) follow-up of 1.76 (2.15) years after ICI, outcomes are: no evidence of disease (2), residual metastatic disease (1), death due to cancer (1), death due to PML (1), and lost to follow-up (2). Notably, 0 out of 7 patients experienced an MS relapse; two out of six had new asymptomatic demyelinating magnetic resonance imaging lesions. In the three patients with expanded disability status scale (EDSS) scores at baseline and follow-up, EDSS remained stable (mean delta 0.13).

**Conclusion:**

In this cohort, no people with MS experienced clinical relapses and one-third experienced asymptomatic radiological activity following ICI treatment.

## Introduction

Population-based studies demonstrate that the incidence of any cancer in people with multiple sclerosis (MS) is ∼4.39%,^1^ suggesting that many people with MS will experience a diagnosis of cancer and make decisions about cancer treatment. Immune checkpoint inhibitor (ICI) therapies, including PD-1, PD-L1, and CTLA-4 inhibitors, have been approved for several different types of cancer including melanoma, lung, bladder, and triple-negative breast cancer^2–4^ and recently have also been used for the treatment of progressive multifocal leukoencephalopathy (PML), an often fatal encephalitis caused by John Cunningham (JC) virus reactivation.^5,6^ These immunotherapies have a high risk of immune-related adverse events (irAEs), with ∼1% to 2% of irAEs representing neurologic side effects.^7^ Side effects involving the central nervous system (CNS) are less common, but frequently severe, including case reports of de novo CNS demyelination.^8–10^

Given the risk of irAEs in patients without autoimmune disease, there is a concern for increased frequency or severity of irAE in patients with pre-existing autoimmune disease. Previous work has reviewed the effects of ICIs on patients with autoimmune diseases, including MS patients, but MS-specific outcomes, including MS relapses, MS disease progression, and MS disease-modifying therapy (DMT) management during ICI therapy are not always included. Most published case reports and case series describing ICI use in MS patients represent negative outcomes; however, it is unclear if these negative outcomes are generalizable to the broader MS population, since case reports may overestimate incidence due to publication bias.

The goal of this study was to characterize clinical outcomes in people with MS who received ICIs at our center in order to provide additional reports about the course of disease in patients with pre-existing autoimmune disease, with a focus on outcomes and management decisions specific to MS.

## Methods

### Study design

The study was a single-center retrospective review of prospectively collected data on patients with MS and exposure to an ICI agent before January 2023. The patient cohort was determined utilizing the Center's electronic health record (EHR) database, EPIC. Criteria for data extraction included a diagnosis of MS, ascertained using a text-based search algorithm modified from prior^11^ that includes but is not exclusive to ICD9 codes, as well as the use of one of the following medications: pembrolizumab, atezolizumab, nivolumab, ipilimumab, avelumab, and durvalumab. Sixteen patients were identified, however, on chart review, only seven patients carried a diagnosis of MS. Four patients were followed at the University of California, San Francisco MS clinic, and one patient was followed at Stanford MS clinic. The other two patients were followed at outside unknown center—for one of the outside patients, the note written by the admitting inpatient physician described that the patient carried a diagnosis of MS, the known presence of brain lesions characteristic of demyelinating disease, and history of DMT therapy; for the second of the outside patients, a non-neurology outpatient specialist noted the patient carried a diagnosis of MS, the year of diagnosis, the known presence of brain lesions characteristic of demyelinating disease, and history of DMT therapy. The remaining nine patients, who were excluded from further analysis, had MS erroneously listed as a past medical problem (e.g. patient had a different neurological diagnosis but a medical provider entered it incorrectly into the EHR), were seen for non-MS-related neurologic issues in the UCSF MS and Neuroinflammation Clinic or by a provider who worked in that clinic or received a medication associated with MS in the EPIC search algorithm (e.g. steroids). Of note, data for one patient included in this series were previously reported in a study focused on breast cancer and MS.^12^

### Clinical variables

Patient records were manually reviewed by two investigators using a standardized data collection form. Demographic data included sex, age at initiation of ICI therapy, as well as race and ethnicity as reported by the patient during initial registration. If a patient declined to provide race or ethnicity or it was unknown, it was marked as unknown/declined. MS type, duration of MS disease at the time of ICI use, MS DMT at time of ICI use, prior MS DMT, and any changes to DMT near the time of ICI use. Information was also extracted about the type of ICI, disease for which ICI was indicated, type and stage of disease, date of diagnosis, time until ICI was used, and other associated treatments for the disease.

### Clinical events and outcomes

Clinical relapses were determined by the treating neurologist as a new neurologic symptom consistent with a demyelinating event and associated with a change in the neurologic exam and/or new enhancing lesion on magnetic resonance imaging (MRI) brain or spine evaluation, lasting at least 24 h and occurring at least 1 month after any prior relapse, and in the absence of infection, fever, or other explanation. If expanded disability status scale (EDSS) scores were not listed by the treating neurologist, they were calculated by an MS-trained neurologist based on recorded neurological exam and reported cognitive symptoms, bladder/bowel function, and ambulation.^13^ If complete information regarding these measures was not available, EDSS scores were not calculated. MRI brain and spine reports after checkpoint inhibitor were reviewed for changes from baseline imaging and categorized by type of change noted. Charts were reviewed for evidence of both neurologic and non-neurologic immune adverse events. Finally, the present condition at the time of data extraction in January 2023 and the duration of follow-up since checkpoint inhibitor use were recorded.

### Statistical analysis

Descriptive analyses were used to characterize these patients and are reported as mean with standard deviation, percentages, or median value (range) for continuous variables. A survival/time-to-event analysis was performed for mortality using Kaplan–Meier analysis, with right censoring.

## Results

### Demographics

Between February 2015 and November 2022, seven patients with MS were treated with ICI. The clinical and demographic characteristics of these patients are described in Table 1. Of these, three were female, and the mean age at ICI use was 55.4 years (SD 13.7). Patients were predominantly White and not Hispanic/Latino.

**Table 1.** Demographic characteristics and outcomes of seven patients with MS who were treated with a checkpoint inhibitor.

Code	Age	Sex	Race	Ethnicity	MS type	MS duration	MS treatment at cancer/PML diagnosis	Change of MS treatment at cancer/PML diagnosis	Disease type	Cancer stage	Immunotherapy	MS relapses	MRI outcomes	Non-neuro irAE	Length of follow-up (years)	Present oncologic condition
1	66	F	White	Not Hispanic/Latino	SPMS	22	Fingolimod	No	Melanoma	4	Pembrolizumab	No	Demyelinating lesions without relapse	Colitis	6.3	No evidence of disease
2	71	M	White	Not Hispanic/Latino	SPMS	34	None	No	Melanoma	3	Ipilimumab, nivolumab	No	No change	None	2.1	Residual metastatic disease
3	61	M	White	Not Hispanic/Latino	MS*	2	None	No	Melanoma	4	Nivolumab, ipilimumab, pembrolizumab	No	No change	Immune-mediated hepatic injury	2.1	Unknown
4	64	F	White	Not Hispanic/Latino	SPMS	30	Ocrelizumab	Yes, held	Breast cancer	2A	Pembrolizumab	No	Demyelinating lesions without relapse	None	1.3	No evidence of disease
5	47	F	Other	Hispanic/Latino	RRMS	15	Natalizumab	Yes, held	PML (natalizumab)	n/a	Pembrolizumab	No	Worsening PML	None	0.4	Deceased
6	39	M	Other	Not Hispanic/Latino	MS*	3	Tecfidera	No	Lung cancer	4	Atezolizumab	No	n/a	None	0.2	Deceased
7	37	M	White	Unknown	RRMS	19	None	No	Melanoma	3B	Nivolumab	No	Brain metastases	Dermatitis	0.1	Unknown

MS: multiple sclerosis; PML: progressive multifocal leukoencephalopathy; irAE: immune-related adverse event; RRMS: relapsing-remitting MS; SPMS: secondary progressive MS.

*No MS subtype was identified from the chart.

### Disease characteristics

Most of the patients received ICIs for oncologic treatment (four melanoma, one lung cancer, and one breast cancer), but one patient was diagnosed with PML and received pembrolizumab as part of the treatment strategy. Of patients who had a diagnosis of cancer (*n* = 6), the median stage at diagnosis was 3.5 (interquartile range 2.75–4); five had metastatic disease at the time of checkpoint inhibitor use, and the sixth received treatment for stage 2A triple-negative breast cancer.

### MS characteristics

MS type was categorized as: “MS” (*n* = 2), secondary progressive (*n* = 3), and relapsing-remitting (*n* = 2). At the time of checkpoint inhibitor use, MS duration was 18.2 years (SD 12.2). Six of the seven patients had exposure to MS DMT at some point during their MS course, and four patients were on treatment at the time of cancer/PML diagnosis (*n* = 1 each for fingolimod, dimethyl fumarate, natalizumab, and ocrelizumab). Two of these four discontinued DMT prior to ICI use, including the patient with natalizumab-associated PML.

### Immunotherapy

Five out of seven patients (71.4%) received pembrolizumab, three patients (42.8%) received nivolumab, two patients (28.5%) received ipilimumab, and one patient (14.3%) received atezolizumab. Notably, two patients received more than one ICI. One patient received ipilimumab and nivolumab, and one patient received ipilimumab, nivolumab, and pembrolizumab; both of the patients receiving multiple ICIs were being treated for melanoma.

### Disease outcomes

At the time of data extraction, with a median time of follow-up of 1.27 years after initiation of ICI, oncologic outcomes were: no evidence of oncologic disease (*n* = 2), residual metastatic disease (*n* = 1), deceased (*n* = 1 due to cancer, *n* = 1 due to PML), and two patients had been lost to follow up. Both deaths were related to complications of the underlying disease and were not attributed to complications of ICI therapy.

### Relapses and events

**Figure 1.**
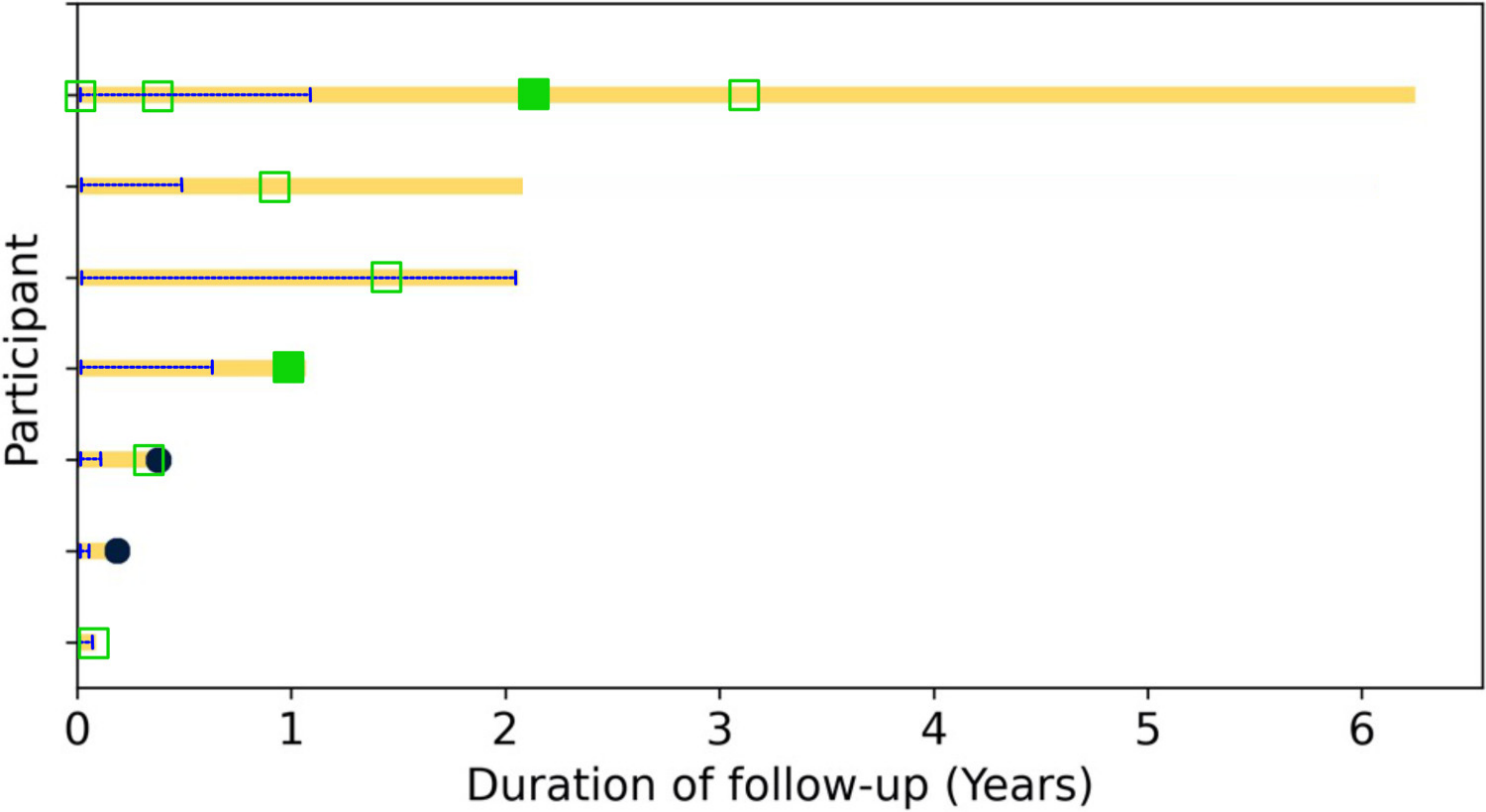
Swimmer plot after ICI exposure. Dark blue circles represent deaths, yellow squares represent censored data points. Dashed blue line demonstrates ICI exposure. Filled green squares represent MRI showing demyelinating lesion, and empty green squares represent MRI without demyelinating lesion. No MS relapses were observed.

ICI: immune checkpoint inhibitor; MRI: magnetic resonance imaging; MS: multiple sclerosis.

**Figure 2.**
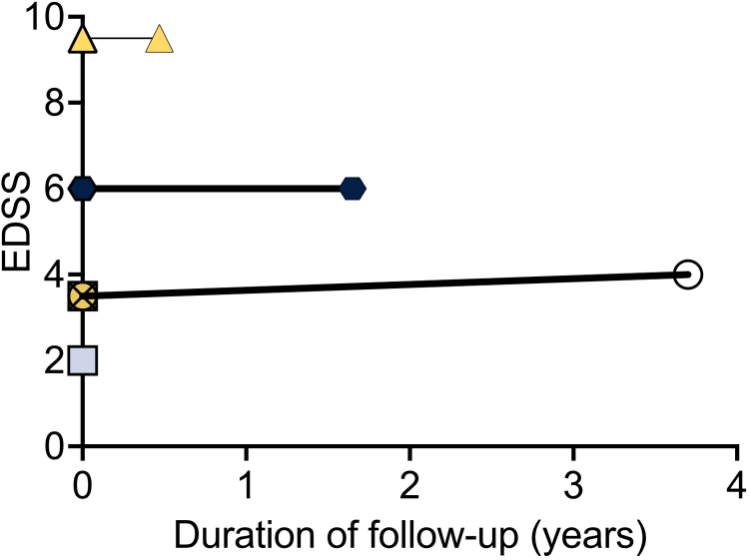
EDSS scores remained stable over the course of follow-up. Each symbol represents a different patient. Solid lines indicate the trend from EDSS scores before and after ICI exposure.

EDSS: expanded disability status scale; ICI: immune checkpoint inhibitor.

No patient experienced an MS relapse or an inflammatory event attributed to immunotoxicity (Figure 1). One patient experienced a pseudo-relapse attributed to recrudescence in the setting of other cancer therapies (allodynia, sense of imbalance). Six patients had MRIs after starting ICI therapy (mean time to MRI after ICI 1.04 years, SD 0.8): only two out of six patients had new asymptomatic demyelinating lesions noted. Additionally, one patient had evidence of metastatic disease, and one patient had worsening PML. Three patients had EDSS scores at baseline and follow-up—these were stable (mean EDSS change 0.13, SD 0.25, mean follow-up 2.02 years, median baseline EDSS 6, and median follow-up EDSS 6), and none experienced a clinically significant change in EDSS (Figure 2). Three of the seven patients experienced non-neurologic irAE: one patient experienced diarrhea that resolved with prednisone and infliximab (without evidence of colitis on endoscopy and colonoscopy), one patient experienced immune-mediated liver injury (treated with high dose steroids and eventually mycophenolate mofetil), and one patient experienced a mild skin rash, thought to potentially represent dermatitis secondary to immunotherapy.

## Discussion

Under physiological conditions, immune checkpoints are critical for maintaining self-tolerance and preventing the development of autoimmunity; however, in the setting of cancer and chronic infections, the persistence of antigen can lead to T cell exhaustion, a dysfunctional state with impaired effector function and cytotoxicity and upregulation of inhibitory molecules such as PD-1 and CTLA-4.^14,15^ ICIs block the receptor–ligand interactions of these inhibitory molecules, resulting in a rejuvenation of the immune response and a more robust anti-tumoral or anti-infectious response. Based on the mechanism of action and published reports, there is a known risk of autoimmune side effects in people exposed to these drugs, and a reasonable concern people with pre-existing autoimmune disease will experience flares or worsening of that disease. Given the prevalence of cancer and the increasing use of ICI therapies for cancer treatment, increasing numbers of people with MS and their physicians will need to consider the use of these therapies and will have questions regarding the incidence and severity of adverse events specific to their disease. In contrast to many prior reports, no people with MS in this cohort experienced new MS clinical relapses attributable to treatment with ICIs, despite only two out of seven being treated with DMTs at the time of the ICI exposure. Additionally, only two patients had new asymptomatic demyelinating lesions (one patient potentially in the setting of DMT discontinuation), and no clinically significant EDSS worsening was observed. While these findings could be due to an older population in whom less inflammatory activity is expected, this could also be a feature of the single-center study design rather than the case report.

To date, understanding of neurological sequelae of ICIs has focused on observations of CNS demyelination in three populations: (a) people without pre-existing autoimmunity or known risk factors, (b) people with risk factors for MS, and (c) people with pre-existing autoimmunity. Many case reports have reviewed adverse events in people without known risk factors for CNS autoimmunity^8–10,16^; in people with apparently new CNS autoimmune demyelination, it is speculated that ICI therapy lowers the threshold for immune activation, leading to a loss of self-tolerance and an “unmasking” of demyelinating disease. This may be further amplified in people with an increased risk of developing MS, for example, due to genetic susceptibility in people with a family history of MS or other autoimmune diseases; additionally, the unmasking of the disease may be more likely after ICI treatment in people with prior brain lesions suspicious for demyelinating disease, whether or not it was diagnosed as radiologically isolated syndrome at the time.^17–20^

In individuals with established diagnosis of MS, however, outcomes after ICI treatment have been confounded by the publication bias of positive findings. For example, an analysis of the FDA adverse event reporting system described 14 cases of new relapse activity in MS patients treated with ICIs.^21^ Severe relapses after ICI were reported in two MS patients treated for melanoma with ipilimumab,^22^ in one patient with MS treated with atezolizumab for non-small cell lung cancer,^23^ and in one patient with retrospectively diagnosed MS treated with nivolumab for lung adenocarcinoma.^24^

In case series and systematic reviews examining relapses in autoimmune diseases more broadly (i.e. including but not exclusive to MS), 27% to 75% of patients with pre-existing autoimmune experienced irAE or exacerbation of their underlying autoimmune, condition; overall, despite the reported increased risk of clinical relapses, these episodes were overall mild.^25–29^ Among the small numbers of MS patients followed in these studies, the frequency of MS relapses ranged between 0% and 18%. In two recent series specifically examining outcomes in MS patients, one out of 27 patients in the two combined studies experienced a relapse, although notably, these were older patients with less inflammatory disease.^30,31^ Larger, multi-center studies will be necessary to determine the true incidence of demyelinating exacerbations in this population, but these small single-center retrospective studies suggest that ICI-associated demyelinating exacerbations in people with pre-existing MS may be less common than exacerbations of pre-existing non-MS autoimmune diseases and that exacerbations in people with MS often respond well to standard treatment of relapses. Additionally, in both this study and prior studies, non-neurologic irAEs are more common than neurologic irAEs in both MS and non-MS populations treated with ICIs. Given that ICIs are typically being considered as part of a treatment plan for people with high-grade cancers with a poor prognosis, they should continue to be considered as an option for people with MS, with the additional consideration of continuing DMT to prevent autoimmune rebound disease. It is already established that stopping certain DMTs, specifically natalizumab and fingolimod, carries the risk of rebound disease activity, however, it remains uncertain whether this risk is heightened when the DMT is discontinued in close proximity to initiating ICI treatment.

This study included one individual treated with pembrolizumab for natalizumab-associated PML, which has demonstrated some success as a treatment option, including in fingolimod-associated PML.^5,6,32^ Given the severity of this condition, despite the potential risk of autoimmunity associated with pembrolizumab, it was nonetheless indicated in order to prevent mortality; unfortunately, at the time of ICI use, the patient was already extremely ill and ultimately succumbed to the illness. To our knowledge, this is the first report of a patient with natalizumab-associated PML treated with pembrolizumab.

The limitations of this study include its small size with heterogeneity of the underlying MS type (and PML) and oncologic disease, retrospective nature, and the relatively short duration of follow-up (2 years). Since this population was predominantly White-non Hispanic and seen at an MS referral Center, findings may not be generalizable to all individuals with MS exposed to ICIs.

## Conclusions

In this single-center cohort, no MS patients experienced clinical MS relapses and one-third experienced asymptomatic radiological activity in the year following ICI treatment. As these drugs become an increasingly common part of cancer treatment, use in MS patients should continue to be cautiously considered.
